# Community and species-specific responses of plant traits to 23 years of experimental warming across subarctic tundra plant communities

**DOI:** 10.1038/s41598-017-02595-2

**Published:** 2017-05-31

**Authors:** Gaurav Baruah, Ulf Molau, Yang Bai, Juha M. Alatalo

**Affiliations:** 1 Department of Evolutionary Biology and Environmental Studies, University of Zurich, Winterthurrerstrasse 190, CH-8057 Zurich, Switzerland; 2 Department of Biological and Environmental Sciences, University of Gothenburg, PO Box 461, SE-405 30 Gothenburg, Sweden; 3 Xishuangbanna Tropical Botanical Garden, Chinese Academy of Sciences, Menglun, Mengla, Yunnan 666303 China; 4 Department of Biological and Environmental Sciences, College of Arts and Sciences, Qatar University, P.O. Box 2713 Doha, Qatar

## Abstract

To improve understanding of how global warming may affect competitive interactions among plants, information on the responses of plant functional traits across species to long-term warming is needed. Here we report the effect of 23 years of experimental warming on plant traits across four different alpine subarctic plant communities: tussock tundra, Dryas heath, dry heath and wet meadow. Open-top chambers (OTCs) were used to passively warm the vegetation by 1.5–3 °C. Changes in leaf width, leaf length and plant height of 22 vascular plant species were measured. Long-term warming significantly affected all plant traits. Overall, plant species were taller, with longer and wider leaves, compared with control plots, indicating an increase in biomass in warmed plots, with 13 species having significant increases in at least one trait and only three species having negative responses. The response varied among species and plant community in which the species was sampled, indicating community-warming interactions. Thus, plant trait responses are both species- and community-specific. Importantly, we show that there is likely to be great variation between plant species in their ability to maintain positive growth responses over the longer term, which might cause shifts in their relative competitive ability.

## Introduction

Recent anthropogenic global warming is likely to pose a major threat to biodiversity^1^. Global warming is predicted to be one of the main drivers of future species extinction^2^. Warming may alter species interactions and could thereby cause local extinction of species^3^. Significant changes in community structure can occur due to warming mediated through changes in plant interactions and growth^4^. Plant distribution, abundance, and phenology are also likely to be significantly affected by climate change in the Arctic^5, 6^. From the Arctic tundra to tropical regions, changes in plant diversity and community structure have already been reported^7, 8^. Particularly, in Arctic tundra, studies have reported that there has been a shift from non-woody to woody vegetation^9, 10^.

Species functional traits and interactions with other species are fundamental in driving community assembly^11, 12^. To successfully predict future changes in community structure, a critical step is to determine how the current anthropogenic changes could affect plant performance and fitness. There is evidence that functional traits directly affect plant physiology and performance, with implications for competitive interactions between plant species^13–16^. Thus in order to predict future changes in species abundance, it is crucial to understand how environmental changes could affect such traits. Recent modelling studies based on plant functional trait and co-occurrence data have shown that unexpected climate-driven community changes can occur, that interactive indirect effects can overcome direct effects and that the timing of species responses is an important driver of community dynamics^17^. Functional trait-based modelling methods have been shown to be more accurate in modelling vegetation distribution and analysing vegetation sensitivity than models built on plant functional type schemes^18^. Such dynamic global vegetation models built on plant functional traits are helpful for assessing vegetation sensitivity to different climatic scenarios. Functional trait plasticity can also be used to asses vulnerability to climate change^19^.

Functional traits define the ecological role of a species and how it interacts with other species and the environment^20^. Environmental perturbation can affect functional traits, which can then affect ecosystem processes^21, 22^. Community dynamics and species abundance have been found to be driven by functional traits that favour rapid growth^23^. Differences in functional diversity can ultimately lead to differences in species abundance over time^23^. Recently, a global study found that three specific functional traits, specific leaf area, wood density and height, are solely responsible for competitive interactions^13^. Plant height and leaf traits including leaf dry matter are important functional response traits that are highly influenced by the abiotic environment, which consequently affects ecosystem properties^21, 24^ (for example by changes in competitive interactions). Moreover, plant traits, especially leaf traits, are sensitive to various environmental variables such as temperature, precipitation sunlight and anthropogenic disturbances like grazing etc.^25, 26^. Plasticity in leaf traits can be critical for species in a rapidly changing environment^27^, as such plasticity would significantly help in a better transient response to a change in the environment than plants with no plasticity. Previous short-term studies have shown that species can respond rapidly in terms of leaf and shoot growth^28, 29^, but that the initial positive responses may not always persist over the longer term^29–31^, suggesting that initial positive short-term responses are poor predictors of longer-term dynamics^31^. Moreover, responses can differ among plant communities^32^, as well as between early and late season^33^. Arctic and alpine regions typically have highly variable climate conditions, both within and between years, so plants can be predicted to respond quickly to favourable conditions when they occur. At the same time, many Arctic and alpine plants are long-lived^34, 35^ and may not be able to allocate limited resources to increased growth over longer periods. Plant growth in Arctic and alpine tundra communities is also frequently nutrient-limited^36^. However, a recent 16-year study on plant traits in five common vascular plant species in three plant communities in Arctic Canada found that tundra plants maintained positive responses in terms of increased leaf size and height after many years of warming^37^, while similar results have been found in a 19-year study in Alaska^38^.

Here we examined the impact of two decades of experimental warming on leaf size (length and width) and plant height of 22 common vascular plant species in four contrasting plant communities above the treeline in subarctic alpine Sweden. We aimed to identify species likely to increase their competitive advantage following long-term warming in terms of increased leaf area and plant height, two of the three functional plant traits responsible for competitive interactions^13^. Another aim was to determine whether within-species responses are consistent across contrasting plant communities. Specifically, we tested the hypothesis that plant traits show positive responses to long-term experimental warming similar to those reported in High Arctic environments.

## Methods

### Study area

The four experimental sites were located at Latnjajaure field station in northern Sweden, at 1000 m elevation in the Latnjavagge valley (68°21′N, 18°29′E) near Abisko. The climate in the area can be classified as subarctic, with cool summers, relatively mild, snow-rich winters and snow cover for most of the year. Mean annual temperature ranges from −1 to −3 °C and total annual precipitation from 600 to 1100 mm. The valley is highly diverse in terms of physical conditions, ranging from dry, nutrient-poor and acidic to wet and base-rich, changes reflected in its plant communities^39, 40^.

### Experimental design

Warming was induced by open-top chambers (OTCs), which increase the temperature by 1.5–3 °C compared with control plots at ambient temperature^39^. Four types of habitats for vegetation communities were selected when assessing the effects of long-term warming on plant traits: dry heath (DH), Dryas heath (Dr), tussock tundra (TT) and wet meadow (WM). In each of these four types of communities, 10 plots with homogeneous vegetation cover were chosen in 1993 and half were assigned to OTC and half to control plots in a pair-wise design. The OTCs were left on plots with warming treatments year-round at all four sites. Detailed information about the plant communities can be found in previous papers^41–44^.

### Plant sampling

At the peak of the growing season (early August 2015), plant height was measured and 10 leaves were sampled, in both the OTCs and control plots, from each of 22 species in the four plant communities included in the study. At sampling, the plots in all four types of communities had experienced 23 years of experimental warming. Leaf length and width were measured on the same day (±1 mm accuracy) in the laboratory at the field station.

### Methods and analyses

To investigate whether warming had significantly affected the selected response variables (leaf length, leaf width, plant height), linear mixed effect model analyses were performed, with treatments (warming, control) and species as fixed effects and individual plants as random effect. For each response variable, normality was assessed using standard diagnostic procedures. If the response variable was normally distributed, the linear mixed effects model was used. Otherwise, a generalised mixed effects model using either Poisson error or Gamma error, depending on how the data were distributed, was applied. Two separate analyses were performed: 1) To determine whether plant traits responded to warming within communities (WM, DH, Dr, TT) and 2) to assess whether plant species responded differently to the treatments among the four different plant communities (WM, DH, Dr, TT). All plant species common to all four communities were pooled together, to study whether the responses in plant traits differed with respect to community.

For the first set of analyses, four different mixed models were evaluated with leaf width, leaf length and plant height as three separate response variables, species and treatment as fixed effects and individual plants as random effect. The best model was selected based on the lowest value of Akaike Information Criterion (ΔAIC)^45^. The different models used to explain the data were: Response variable ~ Treatment * Species; Response variable ~ Treatment + Species; Response variable ~ Treatment; Response variable ~ Species. Not all plant species were present in all the four communities. Hence for the second set of analyses, only the plant species present in at least two communities were selected and pooled, to examine whether there was a difference in the response of the traits to the warming treatment in different communities. For leaf width and length, these species were: *Bistorta vivipara, Salix herbacea*, *Salix reticulata*, *Carex bigelowii* and *Vaccinium vitis-idaea*. For leaf height, the species common to all four communities were: *B. vivipara*, *S. herbacea*, *S. reticulata, C. bigelowii* and *V. vitis-idaea*. Here in these particular analyses, the response variable was the specific species in question (for example, *S*. herbacea). Four different mixed models were evaluated and the best model was selected based on lowest ΔAIC value: Response variable ~ Community * Treatment; Response variable ~ Community + Treatment; Response variable ~ Community; Response variable ~ Treatment. In all these models, random effect of individual plant was included. All analyses were performed using R software^46^.

## Results

### Wet meadow (WM)

**Figure 1:**
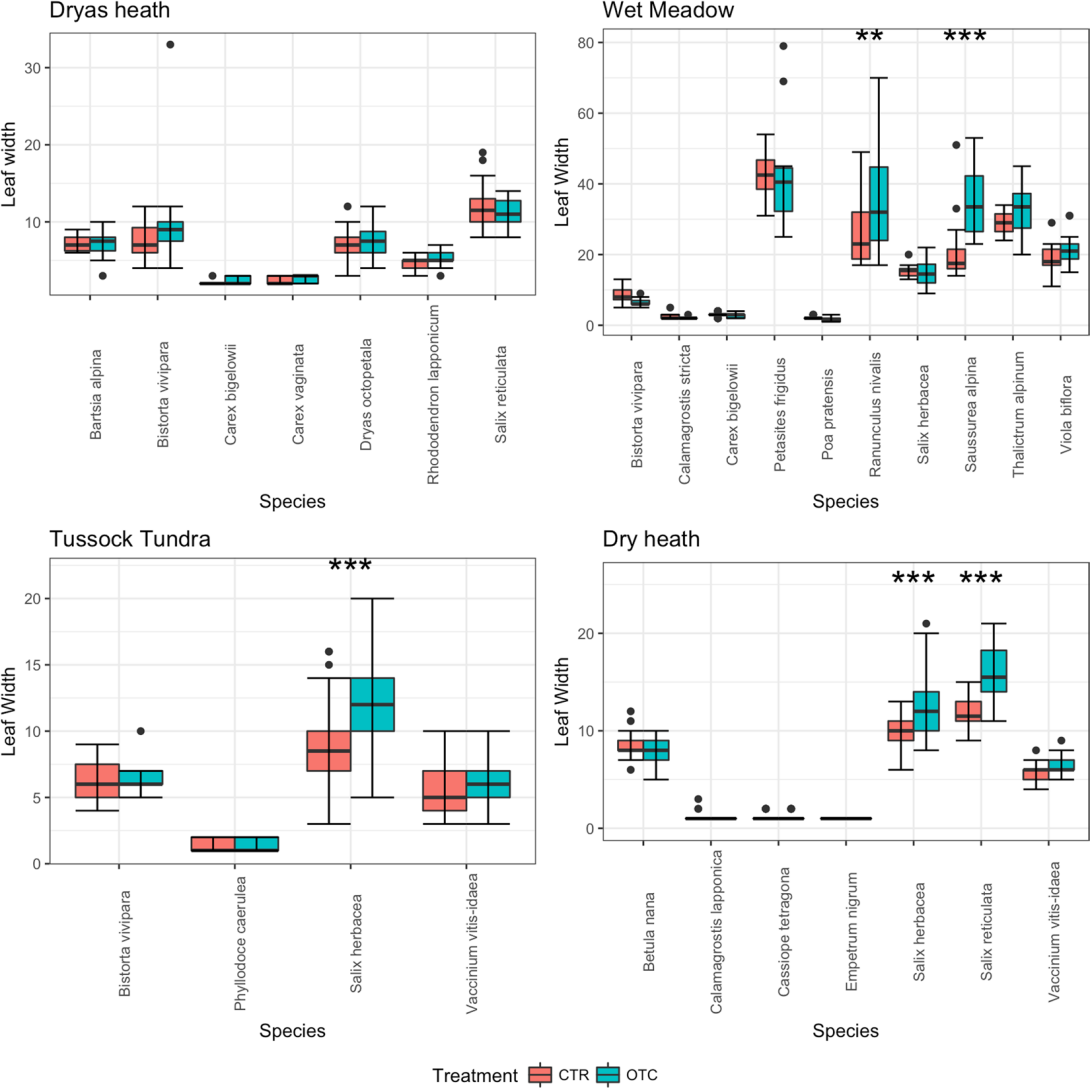
Leaf width (in mm) response of different plant species to 23 years of warming (open-top chamber, OTC) compared with a control treatment (CTR).

**Figure 2:**
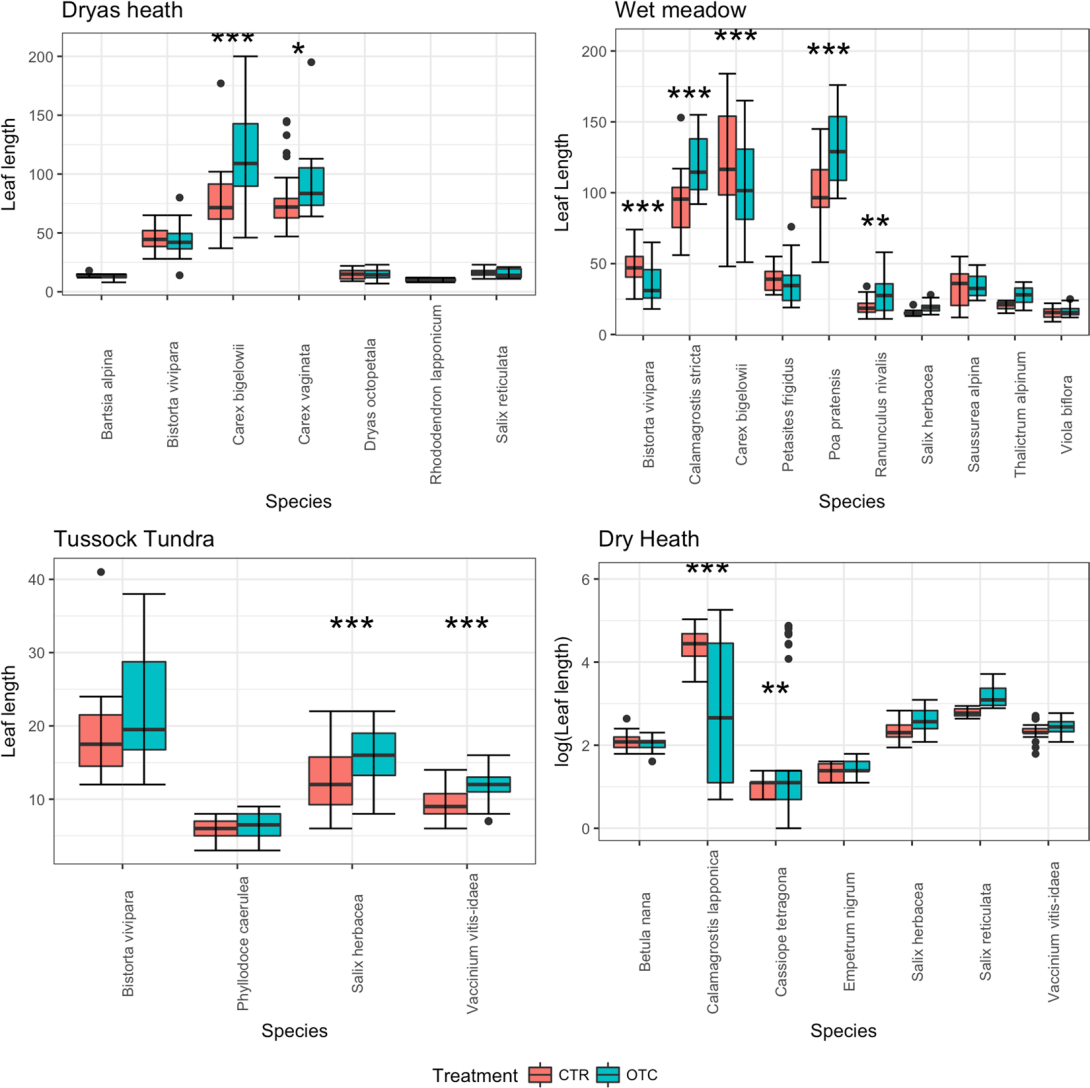
Leaf length (in mm) response of different plant species to 23 years of warming (open-top chamber, OTC) compared with a control treatment (CTR).

For the wet meadow, the best model for leaf width, leaf length and plant height included both fixed effects and their interactions. The response in terms of leaf width varied significantly between species, but warming had significant effects on leaf width in *Ranunculus nivalis* and *Saussurea alpina* (linear comparisons, p < 0.01) (Fig. 1, Table 1). In terms of leaf length, warming had a significant positive effect on *Calamagrostis stricta*, *Poa pratensis* and *R. nivalis* (linear comparisons, p < 0.01) and a significant negative effect on *Bistorta vivipara* and *Carex bigelowii* (linear comparisons, p < 0.01) (Fig. 2, Table 1). The best model for plant height included both the fixed effects of species and treatment and their interaction. Long-term warming had a significantly positive effect on plant height in *R. nivalis, S. herbacea* and *V. biflora*, and a significant negative effect on *B. vivipara, Carex bigelowii* and *P. frigidus* (linear comparisons, p < 0.001) (Table 1). In *Calamagrostis stricta*, *P. pratensis* and *T. alpinum*, there was a non-significant tendency for plants to grow taller in the warming treatment (Table 1).

**Table 1:** Responses in plant species traits (leaf width, leaf length and plant height) to 23 years of warming in the Dryas heath, wet meadow, dry heath and tussock tundra plant communities at Latnjajaure, subarctic Sweden: positive response (+), negative response (−) and no response (0).

Species	Dryas heath			Wet meadow			Dry heath			Tussock tundra		
	Width	Length	Height	Width	Length	Height	Width	Length	Height	Width	Length	Height
*Bartsia alpina*	+	+	na	na	na	na	na	na	na	na	na	na
*Betula nana*	na	na	na	na	na	na	+	+	+***	na	na	na
*Bistorta vivipara*	+	−	+	−	−***	−***	na	na	na	0	+	+
*Calamagrostis lapponica*	na	na	na	na	na	na	na	−***	0	+	na	na
*Calamagrostis stricta*	na	na	na	+	+***	+	na	na	na	na	na	na
*Carex bigelowii*	+	+***	+***	0	−***	−***	na	na	na	na	na	na
*Carex vaginata*	+	+*	+***	na	na	na	na	na	na	na	na	na
*Cassiope tetragona*	na	na	na	na	na	na	na	+**	+	na	na	na
*Dryas octopetala*	+	+	+	na	na	na	na	na	na	na	na	na
*Empetrum nigrum*	na	na	na	na	na	na	0	0	+***	na	na	na
*Dryas octopetala*	+	+	+	na	na	na	na	na	na	na	na	na
*Phyllodoce caerulea*	na	na	na	na	na	na	na	na	na	0	0	0
*Petasites frigidus*	na	na	na	0	0	−***	na	na	na	na	na	na
*Poa pratensis*	na	na	na	0	+***	0	na	na	na	na	na	na
*Ranunculus nivalis*	na	na	na	+***	+**	+***	na	na	na	na	na	na
*Rhododendron lapponicum*	+	+	+	na	na	na	na	na	na	na	na	na
*Salix herbacea*	na	na	na	−	+	+***	+***	+	+***	+***	+***	0
*Salix reticula*	+	+	+	na	na	na	+***	+	+***	na	na	na
*Saussurea alpina*	na	na	na	+***	−	na	na	na	na	na	na	na
*Thalictrum alpinum*	na	na	na	+	+	+	na	na	na	na	na	na
*Vaccinium vitis-idaea*	na	na	na	na	na	na	0	0	+***	0	+***	0
*Viola biflora*	na	na	na	0	0	+***	na	na	na	na	na	na
*Calamagrostis stricta*	na	na	na	+	+***	+	na	na	na	na	na	na
*Calamagrostis lapponica*	na	na	na	na	na	na	na	−***	0	+	na	na
*Eriophorum vaginatum*	na	na	na	na	na	na	na	na	na	na	na	+

Significance lev*e*ls: ***p < 0.0001; **p < 0.001; *p < 0.01; na: data not available.

### Dryas heath (Dr)

**Figure 3:**
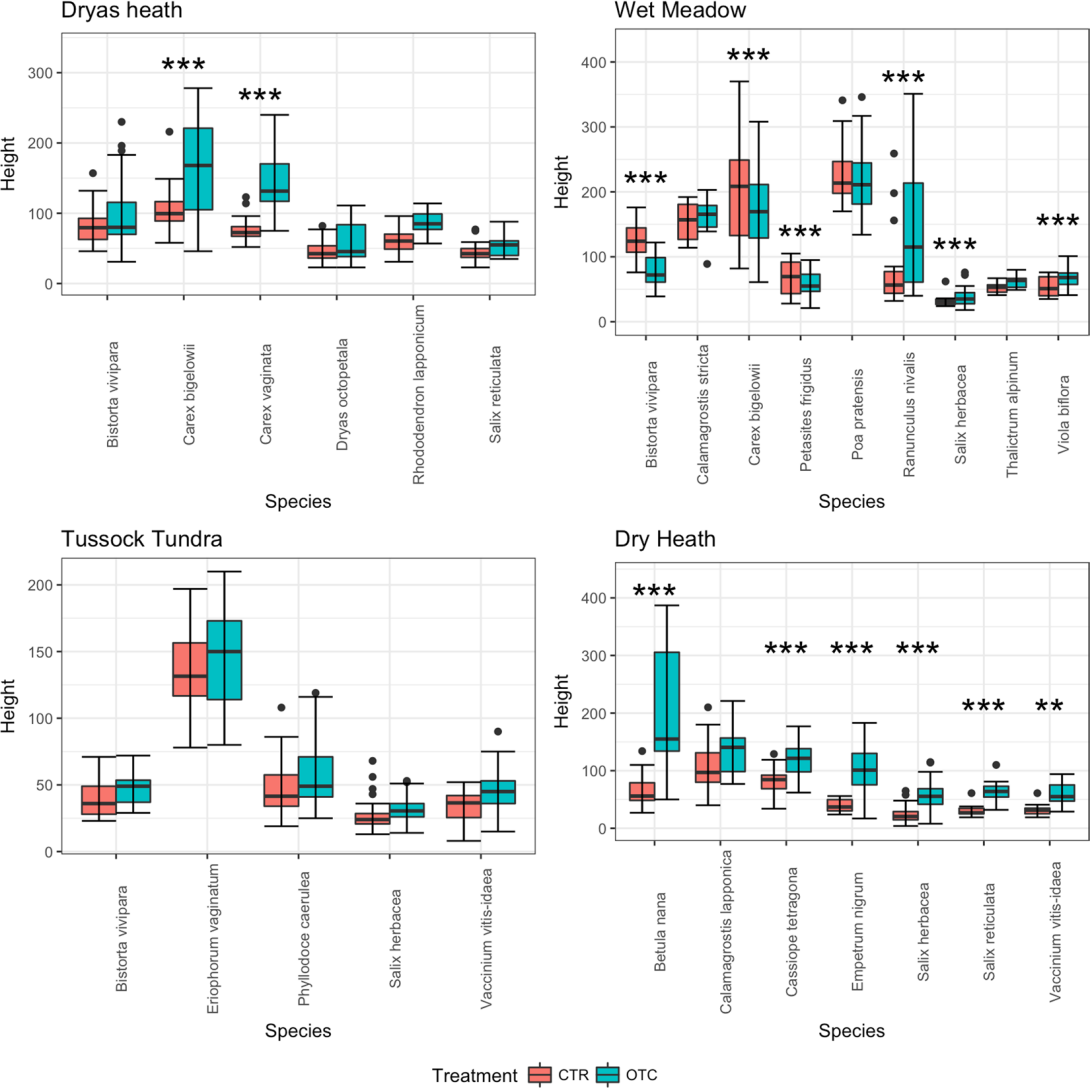
Height response (in mm) of different plant species to 23 years of warming (open-top chamber, OTC) compared with a control treatment (CTR).

For the Dryas plant community, the best model that explained leaf width variation included an additive model of the fixed factors, whereas in the case of leaf length the best model included both the fixed factors and their interactions. As regards the response of leaf width, there was significant variation between all species, but the warming treatment (OTC) in general did not have a significant effect on leaf width compared with the control (CTR), i.e. the interaction of species and warming treatment was not significant. Although *B. vivipara* showed a positive response to warming, this was not significant (Fig. 1, Table 1) (linear comparisons, p = 0.072). In the case of leaf length, the best model included the interaction of the fixed factors. Only *Carex bigelowii* and *C. vaginata* showed a significant positive increase in leaf length in response to warming compared with the control (linear comparisons, p < 0.01) (Fig. 2, Table 1). The best model for plant height included both the fixed effects and their interaction, and long-term warming had a positive effect on *C. bigelowii* and *C. vaginata* (linear comparisons, p < 0.001). Both these *Carex* plant species grew significantly taller in the warming treatment than the plant species in the control plots (Fig. 3, Table 1). Although other species also grew taller in response to warming, the effect of the warming treatment was not significant in these cases (*S. reticula, R. lapponicum*, *D. octopetala, B. vivipara*).

### Tussock Tundra (TT)

For the tussock tundra plant community, the best model for leaf width and for leaf length included both fixed effects and their interaction. In the case of leaf length, there was significant variation among species in response to warming, with only *S. herbacea* and *V. vitis-idaea* (linear comparisons, p < 0.01) having longer leaf length in response to warming compared with the control (Fig. 2, Table 1). In the case of leaf width, there was significant variation between species. However, there was only one species with a significant warming effect: *S. herbacea* (linear comparisons, p < 0.01) (Fig. 1, Table 1). Plant height in the tussock tundra community did not respond significantly to warming (OTC). The best model included the fixed effects and not their interaction term. Although all the species tended to be taller in the warming treatment (*B. vivipara*, *P. caerulea*, *S. herbacea*, *V. vitis-idaea*, *Eriophorum vaginatum*), the response was not significant (Fig. 3, Table 1).

### Dry heath (DH)

For the dry heath community, the best model for leaf width and for leaf length included both the fixed effects and their interaction. In the case of leaf width, although there was significant variation in the response among species, only *S. reticula* and *S. herbacea* responded significantly to warming (OTC) compared with the control (Fig. 1, Table 1). For leaf length, there was significant variation among species, and warming (OTC) had significant positive effect on *Cassiope tetragona* and a significant negative effect on *C. lapponica* (linear comparisons, p < 0.001) (Fig. 2, Table 1). For plant height, the best model included both the fixed effects and their interactions. Warming treatment (OTC) had a significant positive impact on plant height of *Betula nana*, *C. tetragona, E. nigrum, S. reticulata, S. herbacea* and *V. vitis-idaea* (linear comparisons, p < 0.001) (Fig. 3, Table 1).

### Community-specific responses

**Figure 4:**
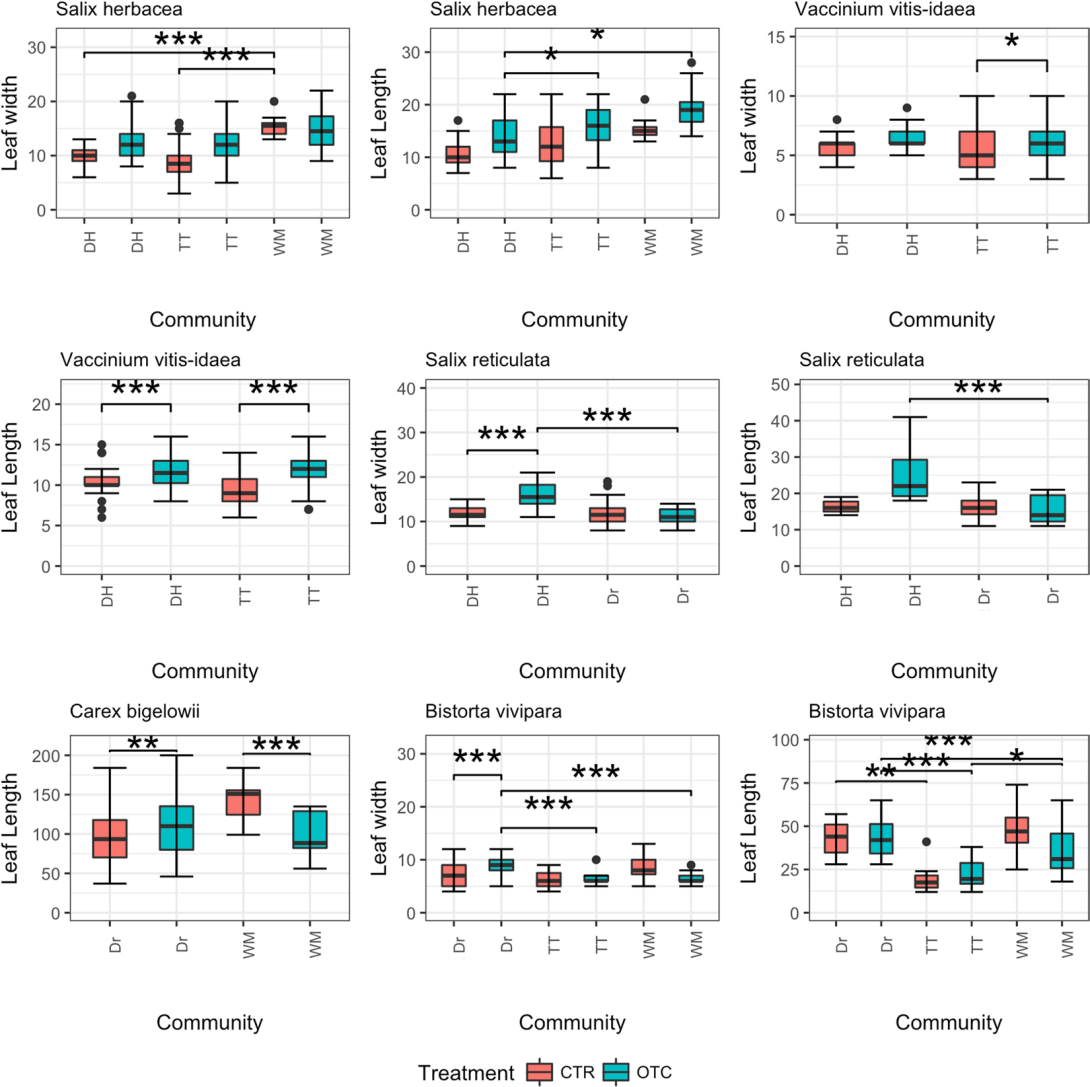
Community-specific response of different plant species to warming in terms of leaf length (in cm) and leaf width (in mm). The six species shown (*Bistorta vivipara, Salix herbacea, Salix reticulata, Carex bigelowii, Calamagrostis stricta, Vaccinium vitis-idaea*) responded to 23 years of warming in at least two communities (Dr = Dryas heath, TT = tussock tundra, WM = wet meadow, DH = dry heath).

Six species (*Bistorta vivipara*, *Salix herbacea*, *S. reticula*, *Carex bigelowii*, *Calamagrostis stricta* and *V. vitis-idaea*) were present in at least two different communities and hence were pooled to see whether there was any community-specific response of plant traits to warming. Except for *V. vitis-idaea*, all these plant species responded significantly differently to warming in different communities (Fig. 4). The best model for all species traits (except *V. vitis-idaea*) included fixed effects of community and treatment and also their interaction. In the case of plant height, the best model for all species (except *S. reticula*) included fixed effects of community and treatment and also their interaction.

#### Carex bigelowii

Linear comparisons of leaf length of *C. bigelowii* in the warming treatment (OTC) between Dryas heath (Dr) and the wet meadow (WM) community did not show any significant differences. There were no effects of warming and community on leaf length of *C. bigelowii*.

**Figure 5:**
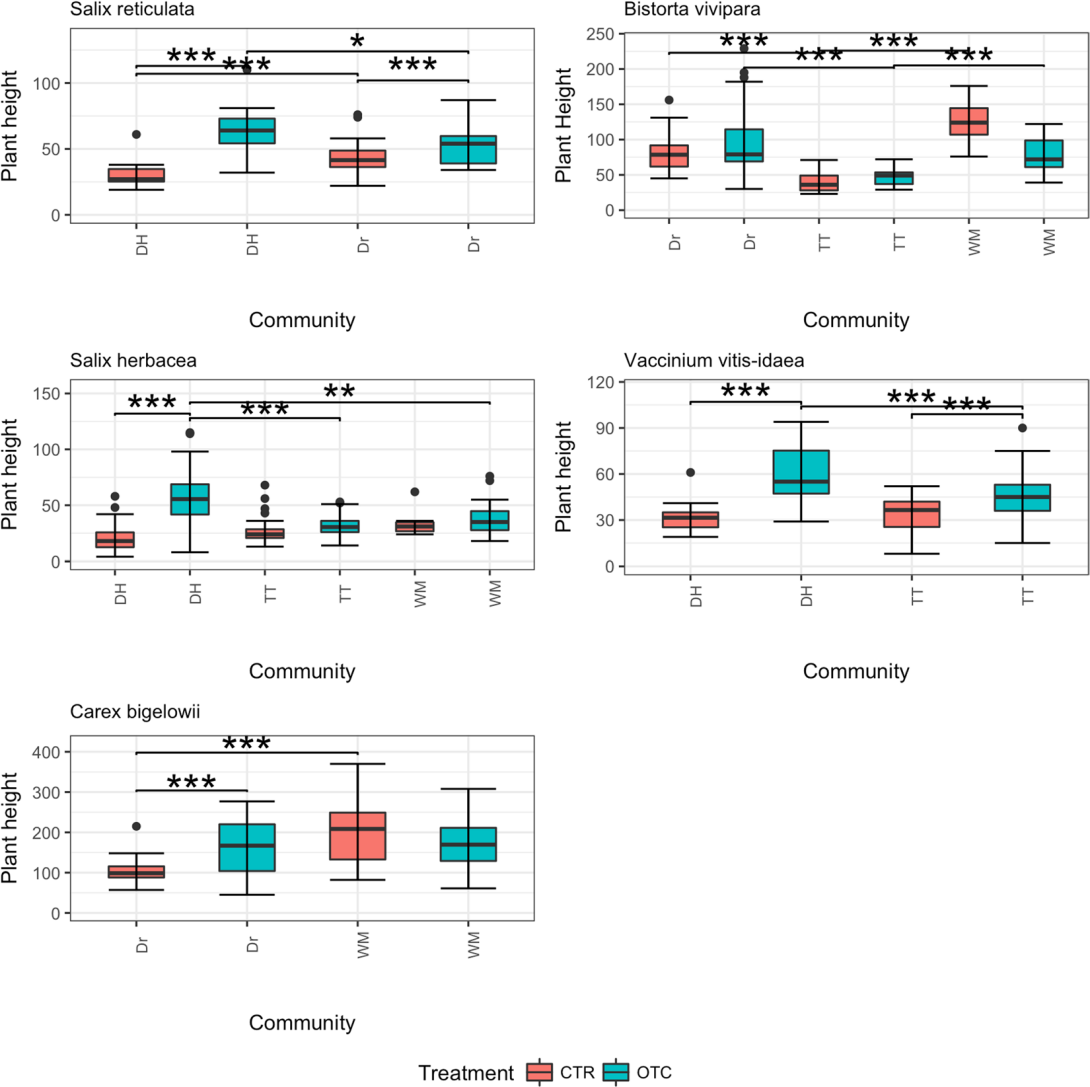
Community-specific response of different plant species to warming in terms of plant height (in mm). The five species shown (*Bistorta vivipara, Salix herbacea, Salix reticulata, Vaccinium vitis-idaea*, *Carex bigelowii*) responded to 23 years of warming in at least two communities (Dr = Dryas heath, TT = tussock tundra, WM = wet meadow, DH = dry heath).

As regards height, plants of *C. bigelowii* in the wet meadow control treatment were significantly taller than those in the Dryas heath control treatment (linear comparison, p < 0.001) (Fig. 5), suggesting there was a community effect. However, on comparing plants of *C. bigelowii* in the warming treatment between the wet meadow and Dryas heath communities, there were no significant differences.

#### Vaccinium vitis-idaea

There were no significant differences in leaf length or leaf width of *V. vitis-idaea* between the dry heath (DH) and tussock tundra (TT) communities in response to warming (OTC) (Fig. 4). However, plant height responded significantly differently in response to warming in dry heath compared with tussock tundra (linear comparisons, p < 0.001) suggesting there was an interaction of warming and community (Fig. 5).

#### Bistorta vivipara

Leaf width in response to warming treatment was significantly smaller in tussock tundra compared with the Dryas heath community (linear comparisons, p < 0.001) and a similar effect was seen for wet meadow (linear comparisons, p < 0.001). However, this was not the case in the control plots (CTR), where leaf length of *B. vivipara* was smaller in tussock tundra than in Dryas heath (linear comparisons, p < 0.001). In warming treatment plots, leaf length was also smaller in wet meadow than Dryas heath (linear comparisons, p = 0.0023), and almost significantly larger in wet meadow than in tussock tundra (linear comparisons, p = 0.06). Leaf length in wet meadow was significantly smaller than in Dryas heath (linear comparisons, p = 0.0012). However, leaf length of *B. vivipara* in the control treatment (CTR) differed significantly between the communities, showing a variable community effect (Fig. 4). There were significant differences in plant height in control plots between Dryas heath and tussock tundra (linear comparisons, p < 0.001) and between tussock tundra and wet meadow (linear comparisons, p < 0.001). This shows that community differences played a role in the response of plant height. Moreover, in the warming treatment plots, there were significant differences in response between Dryas heath and tussock tundra (linear comparisons, p < 0.001). There were also significant differences between tussock tundra and wet meadow (linear comparisons, p < 0.001) (Fig. 5).

#### Salix reticulata

Both leaf width and leaf length of *S. reticulata* in warming treatment plots were significantly larger in dry heath than in Dryas heath (linear comparisons, p < 0.001) (Fig. 4). In the case of plant height, plants of *S. reticulata* in the warming plots were significantly taller in control dry heath than in the warming plots of Dryas heath, suggesting a community-specific effect (linear comparisons, p < 0.05) (Fig. 5).

#### Salix herbacea

Leaf length of *S. herbacea* in warming plots differed significantly between the dry heath, tussock tundra and wet meadow communities (Fig. 4). Leaf length in response to warming treatment was smaller in dry heath than in tussock tundra (linear comparisons, p = 0.03). There was a significant difference in the response of leaf length to warming between wet meadow and dry heath (linear comparisons, p = 0.02), with plants in wet meadow having longer leaves. In contrast, the leaf width response was highly variable across the three communities and no significant differences were seen in response to the warming treatment between the communities. *Salix herbacea* leaves were wider in control plots of wet meadow than in dry heath (linear comparisons, p < 0.001) or tussock tundra (linear comparisons, p < 0.001) (Fig. 4).

Linear comparisons of *S. herbacea* plant height among the control plots of wet meadow, tussock tundra and dry heath showed no significant differences. However, in warming plots, plants in dry heath were significantly taller than those in wet meadow (linear comparisons, p < 0.01) or tussock tundra (linear comparisons, p < 0.001) (Fig. 5).

## Discussion

This study assessed the effects of long-term warming on three plant traits (leaf width, leaf length, plant height) in four types of plant community in subarctic Sweden. The most common responses to long-term warming observed were increased plant height, greater leaf length and greater leaf width. Similar results have been reported in a previous long-term study in High Arctic Canada where, after 15 years of warming, plants had larger leaf size and height in general^37^. The changes observed after 23 years of warming in the present study were strikingly similar to the Canadian results. However, the plant trait responses differed among plant communities, indicating that community-specific responses also occurred. Most species tended to be taller and had larger leaf width and length in warmed plots, with 13 out of 22 (59%) species showing significant increases in at least one of the traits (*Salix herbacea, S. reticulata, Carex bigelowii, Calamagrostis stricta, Carex vaginata, Betula nana*, *V. vitis-idaea*, *P. pratensis*, *Cassiope tetragona*, *R. nivalis*, *Saussurea alpina*, *V. biflora*, *E. nigrum*), and three species having significantly negative responses in at least one trait (*B. vivipara*, *P. frigidus* and *Calamagrostis lapponica*).

However, not all responses were similar, e.g. *Carex bigelowii* traits differed in the Dryas heath (Dr) and wet meadow (WM). Warming had no significant impact on plant height and leaf length in the Dryas heath (Dr) community, but had a negative effect in the wet meadow (WM). This is in line with findings in other studies of contrasting effects of warming among sites^30^. *Carex bigelowii* has also been shown to be unable to maintain initial positive growth responses consistently over a longer time in other studies^30^. The failure of initial positive short-term growth responses in different species to persist over longer periods has been interpreted as possible resource depletion^29–31^. *Bistorta vivipara* showed significant decreases in leaf height and length in wet meadow, but not in the other plant communities in which it was present. Although there were increases in *B. vivipara* leaf width and length in two of the plant communities (wet meadow, dry heath), these were not significantly different from the control plots. After 23 years of warming, *V. vitis-idaea* had significantly taller and longer leaves, but this response was plant-community specific. Similarly, warming had a positive effect on *S. herbacea*, which produced significantly larger leaves and significantly taller plants under long-term warming. However, as in previous studies^37, 47^, plant traits varied significantly between plant communities. In dry heath, plants were significantly taller than in the other habitats, indicating a community-specific response. In tussock tundra, most species showed an increase in leaf size when compared with the control plots, although only two species, *S*. *herbacea* and *V*. *vitis-idaea*, showed significant increases in leaf size. This level of heterogeneity may be due to community-specific responses. While plant species in tussock tundra tended to be taller than in control plots, the change in plant height was not significantly different from the control plots. In wet meadow, leaf width and length were significantly larger than in the other plant communities. *Dryas octopetala* responded to warming with larger leaf width and length and was also taller, as found in other studies^37, 42^. *C. tetragona* was significantly taller after 23 years of warming, while *R. nivalis* also responded positively to warming, becoming significantly taller and having wider and longer leaves than in the control plots, as found in a previous study in High Arctic Canada^37^. However, *C. tetragona* has previously been shown to respond strikingly dissimilarly at high and subarctic sites, with nutrient enhancement having a large positive effect in a subarctic context, while warming is more important at High Arctic sites^48^. In fact, short-term data for our site (Latnjajaure) indicated that *C. tetragona* was not temperature-dependent at this subarctic site^49^.

The shifts in leaf traits will change the competitive interactions and might translate into change in community dynamics^29^. For example, height of a plant species might be significantly important on long-term population level competition^50^. Hence, growing taller in response to warming in our study might be indicative of a species gaining a long-term competitive advantage over other species^50^. In many tundra ecosystems, warming has led to an increase in certain plant functional groups with increased height and larger leaves and this has led to decreases in the cover of shade-intolerant species in tundra ecosystems^51^. Thus being taller and having larger leaves amplify competitive interactions^48^ and affect plant functional diversity and community structure^16^.

The results indicate that response to warming not only varies from species to species but also depends on the plant community. There was significant variation in species responses to warming. Although most species showed an increase in the three plant traits studied in response to warming, a few species showed a decrease. It has been suggested that neighbouring competitive effects might decrease as specific leaf area (SLA) increases^13^. However, SLA decreases as leaf size increases due to greater investment in tissue development^52^. Hence, in our study, increases in leaf size (length and width) in response to long-term warming might be indicative of decreases in SLA and hence increases in neighbourhood competitive effects^53^. Moreover, the increases in leaf width or length or plant height differed depending on the plant community. Thus a plant community-warming interaction also played a role. This differential response of plant traits to a plant community-warming interaction was specifically seen in *Salix herbacea*, *S. reticula*, *Carex bigelowii* and *Bistorta vivipara*. Thus future warming will most likely have significant impacts on the growth of plant species, but the responses are also likely to be plant community-specific.

Plant functional traits are directly linked to plant performance^54, 55^, and are also responsible for community assembly and community dynamics^12, 23^. Moreover, competitive interactions are an important component of community assembly and such interactions are mediated through functional traits^13^. This study showed that warming can significantly affect plant traits over the years, which suggests that competitive interactions and community structure might change under long-term warming. This will have important consequences for plant communities. There is growing concern that functional diversity will be affected by the anthropogenic changes that are predicted to occur in future^56^. Plant height and leaf traits are the most important and consistent drivers of ecosystem functioning^57^. These functional traits are linked in particular to plant resource economics, biomass production and soil water retention^57, 58^. Hence the changes in leaf traits and plant height that we observed in our study after 23 years of experimental warming will have direct impacts on ecosystem services and, in particular, biomass production. These findings, which are largely consistent with results in previous studies^37^, imply that trait-based studies should be linked to community studies and ecosystem services, in order to better understand how long-term warming can change the structure of plant communities and ecosystem functioning.

## Electronic supplementary material

Dataset 1

Dataset 2
